# WHO recommends new name for monkeypox disease

**Published:** 2022-12

**28 November 2022** - Following a series of consultations with global experts, WHO will begin using a new preferred term “mpox” as a synonym for monkeypox. Both names will be used simultaneously for one year while “monkeypox” is phased out.

When the outbreak of monkeypox expanded earlier this year, racist and stigmatizing language online, in other settings and in some communities was observed and reported to WHO. In several meetings, public and private, a number of individuals and countries raised concerns and asked WHO to propose a way forward to change the name.

Assigning names to new and, very exceptionally, to existing diseases is the responsibility of WHO under the International Classification of Diseases (ICD) and the WHO Family of International Health Related Classifications through a consultative process which includes WHO Member States.

WHO, in accordance with the ICD update process, held consultations to gather views from a range of experts, as well as countries and the general public, who were invited to submit suggestions for new names. Based on these consultations, and further discussions with WHO’s Director-General Dr Tedros Adhanom Ghebreyesus, WHO recommends the following:

Adoption of the new synonym mpox in English for the disease.

Mpox will become a preferred term, replacing monkeypox, after a transition period of one year. This serves to mitigate the concerns raised by experts about confusion caused by a name change in the midst of a global outbreak. It also gives time to complete the ICD update process and to update WHO publications.

The synonym mpox will be included in the ICD-10 online in the coming days. It will be a part of the official 2023 release of ICD-11, which is the current global standard for health data, clinical documentation and statistical aggregation.

The term “monkeypox” will remain a searchable term in ICD, to match historic information.

Considerations for the recommendations included rationale, scientific appropriateness, extent of current usage, pronounceability, usability in different languages, absence of geographical or zoological references, and the ease of retrieval of historical scientific information.

Usually, the ICD updating process can take up to several years. In this case, the process was accelerated, though following the standard steps.

Various advisory bodies were heard during the consultation process, including experts from the medical and scientific and classification and statistics advisory committees which constituted of representatives from government authorities of 45 different countries.

The issue of the use of the new name in different languages was extensively discussed. The preferred term mpox can be used in other languages. If additional naming issues arise, these will be addressed via the same mechanism. Translations are usually discussed in formal collaboration with relevant government authorities and the related scientific societies.

WHO will adopt the term mpox in its communications, and encourages others to follow these recommendations, to minimize any ongoing negative impact of the current name and from adoption of the new name

Available from: **https://www.who.int/news/item/28-11-2022-who-recommends-new-name-for-monkeypox-disease**

